# The impact of COVID-19 pandemic on hospital admissions for nine diseases in Iran: insight from an interrupted time series analysis

**DOI:** 10.1186/s12962-022-00394-9

**Published:** 2022-11-01

**Authors:** Sina Ahmadi, Ali Kazemi-Karyani, Nasim Badiee, Sarah Byford, Ali Mohammadi, Bakhtiar Piroozi, Satar Rezaei

**Affiliations:** 1 Social Welfare Management Research Center, Department of Social Welfare Managment, University of Social Welfare and Rehabilitation Sciences, Tehran, Iran; 2 Research Center for Environmental Determinants of Health, Health Institute, Kermanshah University of Medical Sciences, Kermanshah, Iran; 3 Department of Traditional Medicine, School of Persian Medicine, Iran University of Medical Sciences, Tehran, Iran; 4 Health Service & Population Research, Institute of Psychiatry, Psychology & Neuroscience, King’s College London, London, UK; 5 Department of Health Information Technology, Paramedical School, Kermanshah University of Medical Sciences, Kermanshah, Iran; 6 Social Determinants of Health Research Center, Research Institute for Health Development, Kurdistan University of Medical Sciences, Sanandaj, Iran

**Keywords:** COVID-19 pandemic, Hospitalization rate, Interrupted time series analysis, Iran

## Abstract

**Background:**

Associations between the COVID-19 pandemic and hospitalizations have not been studied Iran. This study aimed to examine the impact of the COVID-19 pandemic on hospital admissions for nine categories of disease in seven public hospitals in Kermsnahah city, the capital of Kermsnahah province, in the west of Iran.

**Methods:**

Data on monthly hospitalization rates (number of hospitalizations per 100,000 population) were collected for nine categories of disease for a period of 40 months (23 months before and 17 months after the COVID-19 outbreak in Iran) from the health information systems of all seven public hospitals in Kermanshah city. Categories of disease included those related to pregnancy, childbirth and the puerperium period, neoplasms, diseases of the digestive, respiratory, circulatory, genitourinary and nervous systems, mental and behavioural disorders, and infectious and parasitic diseases. Population data were extracted from the Statistics Centre of Iran. An interrupted time series analysis with segmented regression was used to examine the impact of COVID-19 on hospital admissions.

**Findings:**

Average monthly hospitalization rates fell for all nine categories of disease included in the study after the onset of the pandemic, with overall rates of 85.5 per 100,000 population in the period before the COVID-19 outbreak and 50.4 per 100,000 population after the outbreak began. The relative reduction in hospitalizations for the nine diseases was 56.4%. Regression analysis of monthly data indicated a sharp decrease in hospitalisations during the first month after the COVID-19 outbreak, which was statistically significant for all diseases (p < 0.001). After the initial reduction following onset of the pandemic, significant increases were observed for some diseases, including neoplasms (increase of 3.17 per 100,000 population; p < 0.001), diseases of the digestive system (increase of 1.17 per 100,000 population; p < 0.001) and diseases related to pregnancy, childbirth and the puerperium period (increase of 1.73 per 100,000 population). For other categories of disease, rates significantly declined, including infectious and parasitic diseases (decrease of 2.46 per 100,000 population; p < 0.001). Hospitalization rates did not increase to pre-pandemic levels for any disease, with the exception of those related to pregnancy, childbirth and the puerperium period.

**Conclusions:**

Our study indicated that the COVID-19 pandemic had a significantly negative effect on hospitalizations in Iran. Although use of hospital care has gradually increased post-outbreak, it has yet to return to normal levels.

## Introduction

In late December 2019, in Wuhan, Hubei province, China, a coronavirus disease (COVID-19) with unknown causes broke out [1]. COVID-19 spread rapidly and enveloped most countries around the world. On March 11, 2020, the World Health Organisation (WHO) declared COVID-19 a pandemic, the first such declaration since H1N1 in 2009. In Iran, the outbreak of COVID-19 officially began after the first death from COVID-19 was identified in Qom on February 19, 2020 and quickly spread to all provinces of Iran [2]. COVID-19 is one of the most significant global public health concerns and it is the third deadly coronavirus outbreak in less than 2 decades [3]. The number of cases diagnosed with COVID-19 has risen dramatically, with 416,591,750 confirmed cases worldwide and 5,859,567 deaths recorded as of February, 16, 2022 [4]. Thus, extraordinary rigorous public health interventions were implemented, such as quarantines, the wearing of face masks, regular hand washing, stay-at-home orders, and maintaining physical distance [3]. These policies and the outbreak of COVID-19 caused significant disruptions to the daily life of people around the world and also had a significant impact on health services, including the number of hospital visits for other diseases. Health services became overwhelmed by COVID-19 cases, many non-emergency appointments were cancelled and people chose to stay at home rather than risk exposure to the virus. As health systems around the world respond to the coronavirus disease, there is increasing concern that patients with other diseases continue to avoid care [5, 6].

Previous studies [7–11] have shown significant reductions in the number of persons seeking medical care during the COVID-19 outbreak, suggesting the virus is impacting the behavior of entire populations in relation to willingness to accept direct treatment [12]. Hospitals have also reported unexplained reductions in admissions for serious medical illnesses [13]. Reductions in hospitalization rates could indicate significant harm to public health of patients whose treatment for life-threatening diseases is postponed.

An interrupted time-series analysis in China indicated that observed total hospital visits and observed hospital visits for diseases of the respiratory system decreased by 22.60% and 62.25%, respectively, after the end of the COVID-19 lockdown in China [8]. A nationwide study in France found that the number of pediatric emergency department visits decreased by 68% after the outbreak of COVID-19 [7]. A study in the United States demonstrated that during the early pandemic period, the total number of US emergency department visits was 42% lower than during the same period a year earlier, with the greatest reduction in visits by females and persons aged ≤ 14 years [11]. In addition, decreases in recruitment of patients into cancer clinical trials were observed, despite positive steps taken [14]. Other studies suggest that even among hospitals experiencing relatively low numbers of COVID-19 admissions, admissions for other diseases have still fallen [15, 16]. These findings support the argument that people were avoiding going to hospitals because they feared contracting the COVID-19 virus [12].

Although healthcare utilization has decreased during the pandemic in all countries [9, 16, 17], this reduction has varied across different health systems and different diseases. To our knowledge, no study has comprehensively explored the effect of the COVID-19 pandemic on national healthcare service utilization and hospital admissions in Iran. The aim of the present study was to investigate the impact of the COVID-19 pandemic on hospital admissions in Iran and to explore the differential impact for nine categories of disease.

## Methods

### Study setting

This study was conducted in Kermanshah city, the capital of Kermanshah province, which is located in the western region of Iran. In Iran’s health system, hospital services are provided within both public and private sectors. Kermanshah city has seven Ministry of Health and Medical Education-affiliated hospitals that are responsible for the provision of most inpatient services in the city, all of which participated in the present study.

## Data source

Data for the study were collected from the health information systems (HIS) of all seven hospitals using a self-construct check list. The first confirmed case of the Covid-19 outbreak in Iran was reported on the 19th February 2020. Data were collected from all included hospitals on monthly hospitalization rates per 100,000 population for nine disease categories based on ICD-10, including: certain infectious and parasitic diseases (A00–B99); neoplasms (C00–D48); mental and behavioural disorders (F00–F99); diseases of the nervous system (G00–G99); diseases of the circulatory system (I00–I99); diseases of the respiratory system (J00–J99); diseases of the digestive system (K00–K93); diseases of the genitourinary system (N00–N99) and diseases of pregnancy, childbirth and the puerperium period (O00–O99). Data were collected for a total period of 40 months, including 23 months before the COVID-19 outbreak (from 21st March 2018 to 18th February 2020) and 17 months after the Covid-19 outbreak (19th Februray 2020 to 21st July 2021). In addition, population data were extracted from the Statistics Centre of Iran (SCI). We divided the number of hospitalizations per month divided by the population in a year to eliminate the effects of population growth.

### Statistical analysis

**Fig. 1:**
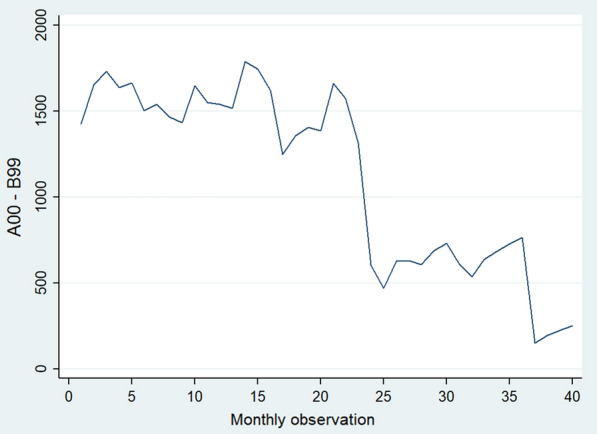
The twoway line grpah for cheking seasonality pattern Certain infectious and parasitic diseases (A00–B99)

In line with previous studies [18, 19], an interrupted time series with segmented regression analysis was used to examine the effect of the COVID-19 outbreak on monthly hospitalization rates per 100,000 population for the nine disease areas included in the study. One of the most important advantages of this approach is that there is no need for a comparison group to examine an association between interventions (in this case, COVID-19) and outcomes (hospitalizations) [19, 20]. In this study we used the the Newey-West approach to deal with autocorrelation and possible heteroscedasticity [19]. Moreover, to ensure that we estimated a model that accounted for the correct autocorrelation structure, *actest* command was used to determine the suitable lag to correct autocorrelation for each of the dependent variables in this study. The following specific segmented regression model was used to estimate the effect of the COVID-19 outbreak on monthly hospitalization rates for included diseases [20, 21]:

$$Y_{t} = \beta _{0} + \beta _{1} *time_{t} + \beta _{2} *{\text{COVID}} - 19_{t} + \beta _{3} *timeafterCOVID - 19_{t} + \varepsilon _{t} ,$$

where ${Y}_{t}$ represents the hospitalization rate in the month $t$, and ${time}_{t}$ is a time trend variable which takes values between 1 (first observation) and 40 (last observation). The ${\text{COVID - }}19_{t}$ variable is a binary variable for before (COVID-19 = 0) and after (COVID-19 = 1) the outbreak. The ${\text{time}}\,{\text{after}}\,{\text{COVID - }}19_{t}$ variable is assigned 0 for the period before the COVID-19 outbreak and coded between 1 and 17 for the period after the COVID-19 outbreak. In the model, ${\beta }_{0}$ estimates the starting level of the outcome variable at time zero, ${\beta }_{1}$ estimates the change in the monthly hospitalization rate before the COVID-19 outbreak (the pre-existing trend), ${\beta }_{2}$ estimates the level of change in the monthly hospitalization rate immediately after the COVID-19 outbreak, and ${\beta }_{3}$ estimates the monthly change in the monthly hospitalization rate trend after COVID-19, compared with the pre-existing trend. We used the augmented Dickey–Fuller test (dfuller command in Stata) to examine the stationary for each of the dependent variables in this study. The output of the test indicated that the p-value for all variables was less than 0.05. in this test, the null hypothesis is always that the variable has a unit root and p-value less than 0.05 means that the studied variable is stationary. For example, the p-value for diseases of the genitourinary system (N00–N99) was 0.0183 and for mental and behavioral disorders (F00–F99) was 0.026 (Table 1). In terms of seasonality, if a cycle structure in a time series constantly repeats at the same frequency, it is seasonal, otherwise it is not seasonal. We checked the seasonality for each of the dependent variables in this study by using the twoway line command. The output graphs indicated that we did not observe seasonality patterns in our data. For example, the result of certain infectious and parasitic diseases (A00–B99) category illustrated in Fig. 1. All data analysis was performed using Stata statistical software (Version 16.0; Stata Corporation, College Station, TX, USA).

**Table 1:** Results of Dickey-Fuller test for unit root in selected disesaes

Dickey-Fuller test for unit root; Number of obs = 39				
	Test Statistic	1% Critical value	5% Critical value	10% Critical value
Diseases of the genitourinary system (N00–N99)^a^	− 3.231	− 3.655^**^	− 2.961^*^	− 2.613^*^
Mental and behavioural disorders (F00–F99)^b^	− 3.103	− 3.655^**^	− 2.961^*^	− 2.613^*^

^a^MacKinnon approximate p-value for Z(t) = 0.0183

^b^MacKinnon approximate p-value for Z(t) = 0.0263

*significant; **insignificant

## Results

Total hospital admissions for the studied diseases, detailed in Table 2, were 275,941 during the study period, 192,117 before and 83,824 after the COVID-19 outbreak. The relative reduction in hospitalization for the nine disease after the outbreak compared to the period before the pandemic was 56.4%. The average monthly hospitalization rate due to all nine categories of disease included in the study was 85.5 per 100,000 population before and 50.4 per 100,000 population after the outbreak (Table 2).

**Table 2:** Description of total and cause-specific hospital admissions during the pre- and post-COVID-19 outbreak

Diseases included in the study (ICD-10)	Before COVID-19 outbreak		After COVID-19 outbreak	
	Total hospitalizations^a^	Average monthly hospitalization rate per 100,000 population	Total hospitalization^b^	Average monthly hospitalization rate per 100,000 population
Certain infectious and parasitic diseases (A00–B99)	35,392	142	9,133	49
Neoplasms (C00–D48)	46,619	187	19,268	104
Mental and behavioural disorders (F00–F99)	5058	20	2214	12
Diseases of the nervous system (G00–G99)	6959	28	2013	11
Diseases of the circulatory system (I00–I99)	27,372	110	13,988	76
Diseases of the respiratory system (J00–J99)	12,375	50	2840	15
Diseases of the digestive system (K00–K93)	16,910	68	8068	44
Diseases of the genitourinary system (N00–N99)	14,845	59	8015	43
Pregnancy, childbirth and the puerperium (O00–O99)	26,587	106	18,285	99

^a^Covers the period 21st March 2018 to 18th February 2020

^b^Covers the period 19th February 2020 to 21st July 2021

The results of the segmented regression models for hospitalization rate for the nine included diseases before and after the onset of the COVID-19 pandemic are reported in Table 3. Regression analysis of monthly data indicated a sharp and statistically significant decrease in the first month after the COVID-19 outbreak for all nine diseases (p < 0.001). The reduction in the first month after the outbreak was highest for neoplasms (88.5 per 100,000 population), followed by infectious and parasitic diseases (58.9), diseases of the circulatory system (39.2), diseases of the respiratory system (37.1), diseases of the digestive system (28.5), diseases of the genitourinary system (22.5), pregnancy, childbirth and the puerperium (18.2), diseases of the nervous system (16.8), and mental and behavioural disorders (12.1).

**Table 3:** Estimated coefficients of segmented regression model for admission rate for nine diseases included in the study before and after COVID-19 pandemic in Iran

Diseases included in the study (ICD-10)	Intercept, *β*_*0*_	Pre-COVID19 pandmic slope, *β*_*1*_	Change in slope, *β*_*2*_	Change in trend, *β*_*3*_	Linear trend, *β*_*p1*_^*a*^
Certain infectious and parasitic diseases (A00–B99)	147.6*	− 0.47**	− 58.9*	− 2.46*	− 2.94*
Neoplasms (C00–D48)	201.9*	− 1.15*	− 88.5*	3.17*	2.01*
Mental and behavioural disorders (F00–F99)	16.4*	0.36*	− 12.1*	− 0.31**	0.05**
Diseases of the nervous system (G00–G99)	24.7*	0.29*	− 16.8*	− 0.54**	− 0.25**
Diseases of the circulatory system (I00–I99)	111.5*	− 0.22**	− 39.2*	1.44**	1.22**
Diseases of the respiratory system (J00–J99)	41.5*	0.76**	− 37.1*	− 1.21**	− 0.45**
Diseases of the digestive system (K00–K93)	70.9*	− 0.26**	− 28.5*	1.17*	0.91*
Diseases of the genitourinary system (N00–N99)	60.1*	− 0.07**	− 22.5*	1.03**	0.96*
Pregnancy, childbirth and the puerperium (O00–O99)	109.0*	− 0.21**	− 18.2*	1.73*	1.52*

^*^Significant at the 5% level

**Not significant

^a^This obtained from the following time trend Equation:${Y}_{pt}= {\beta }_{p0}+ {\beta }_{p1}*{time}_{pt}+{\varepsilon }_{t}$ where ${Y}_{pt}$ is the value of hospitalization rate per 100,000 population at time $t$ after the Covid19 pandemic and ${time}_{pt}$ is the time trend variable which takes values between 1 (first observation after the pandemic) and 17 (last observation after the pandemic)

**Fig. 2:**
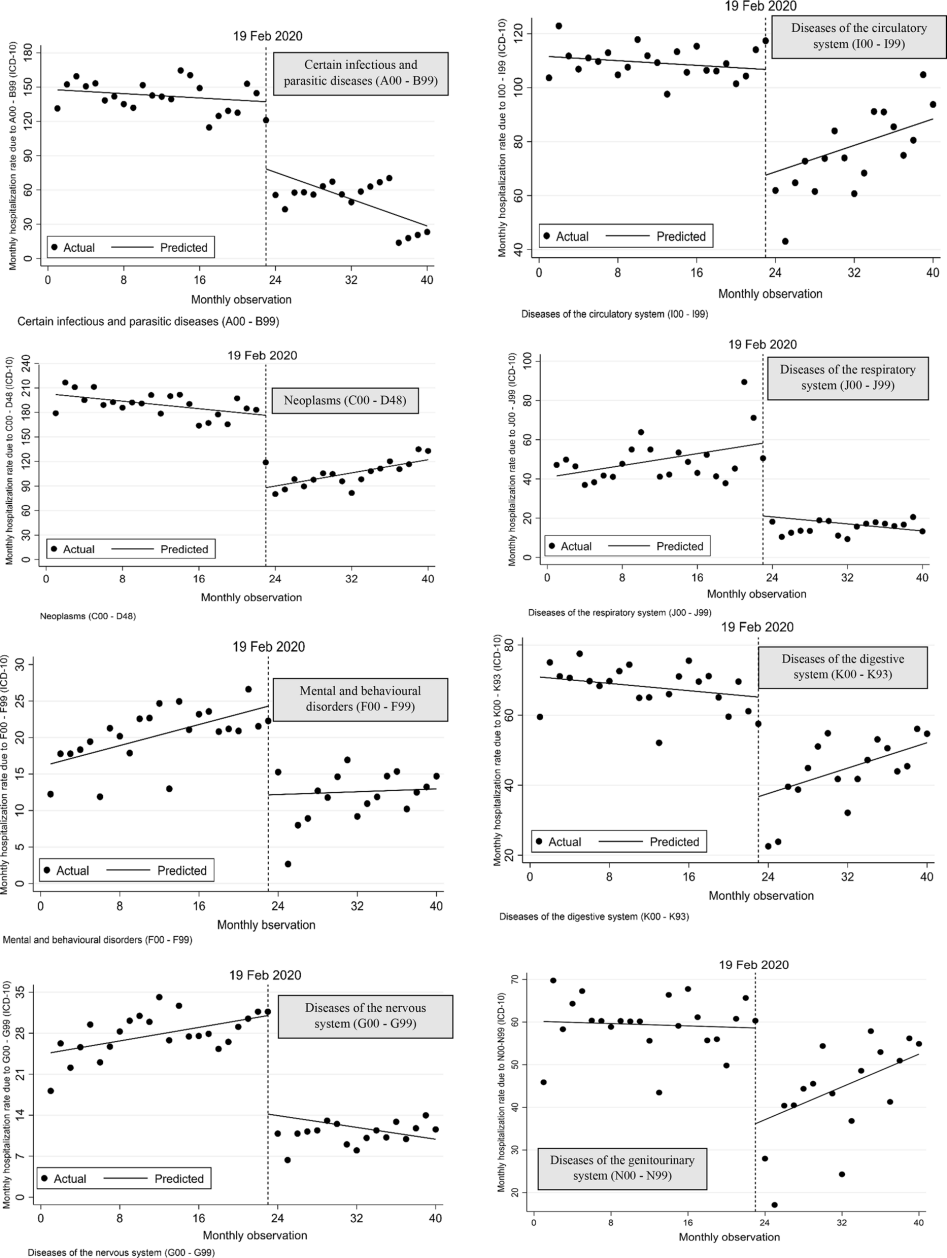

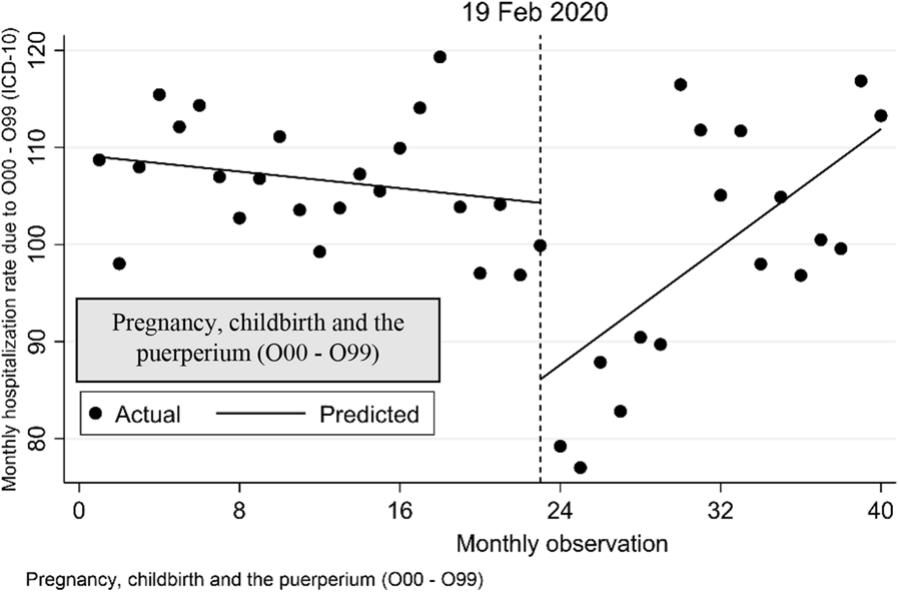
Interrupted time series analysis with Newey–West standard errors and two lags for hospitalizations rate per 100,000 population due to nine diseases included in the study. Note: The pandemic outbreak was in 19 February 2020

Interrupted time series analysis with Newey–West standard errors and two lags for hospitalization rates per 100,000 population for each of the nine diseases included in the study are illustrated in Fig. 2. Compared with the monthly trend in hospitalization rates over the 23 months before the COVID-19 outbreak, over the 17 months after the outbreak began we found a statisitically significant increase in the monthly trend in hospitalizations of 3.17 per 100,000 population for neoplasm disease (p < 0.001), 1.17 for diseases of the digestive system (p < 0.001) and 1.73 for diseases associated with pregnancy, childbirth and the puerperium period. Conversely, we observed a significant dcrease of 2.46 for certain infectious and parasitic diseases (p < 0.001). No significant trends were observed for the remaining disease categories.

## Discussion

Healthcare utilization has repeatedly been observed to decrease during the COVID-19 pandemic [16, 17]. Iran, as one of the countries with a high incidence of COVID-19, was found to be similarly affected, with steep reductions in the rate of hospitalizations, particularly early in the pandemic. It is plausible that a proportion of these reductions in medical admissions were for circumstance that could appropriately be managed at home [22]. However, such reductions in the number of hospital visits may also have dangerous consequences for some patients. It is well established that prompt diagnosis and treatment are important for public health and treatment delay is a predictor of adverse outcome [9].

Our study indicates that hospital admissions fell dramatically with the onset of the COVID-19 pandemic for all of nine non-COVID-19 diseases investigated, including infectious and parasitic diseases, neoplasms, mental and behavioral disorders, diseases of the nervous system, diseases of the circulatory system, diseases of the respiratory system, diseases of the digestive system, diseases of the genitourinary system, and pregnancy, childbirth and the puerperium. Our results demonstrate a sharp and significant decrease in hospitalizations in the first month after the COVID-19 outbreak, which is consistent with previous studies in other countries [6–8, 16, 17]. A recent WHO survey looked at the extent of the disruption of services for the prevention and treatment of non-communicable diseases [23]. One study in China showed that total healthcare expenditure and frequency of healthcare utilization decreased in medium-risk and high-risk cities during and after the COVID-19 outbreak [24]. Furthermore, in a nationwide survey carried out in Italy, De Rosa and colleagues demonstrated that hospitalizations for myocardial infarction during the COVID-19 pandemic were halved compared with the equivalent period of the previous year [13].

Supply-side factors clearly contributed substantially to the sharp decline in hospitalizations and health service utilization for non-COVID-19 diseases. Initial lack of knowledge of the COVID-19 virus and reduced capacity within hospitals due to the substantial number of COVID-19 patients requiring hospital treatment, reduced the ability of hospitals to address non-COVID-19 health disorders, with non-urgent treatments being cancelled and delayed and resources reallocated to fighting the virus [24].

Demand-side factors are also likely to be important, with patients and their family members choosing not to seek treatment or attend hospital for fear of getting infected with the virus [17, 25] or as a result of lockdown restrictions [24]. For example, we observed that the hospitalization rate for certain infectious and parasitic diseases has decreased more than twice in the first month after the pandemic compared to latest months included in the study). A study in Hong Kong [24], for example, indicated that patients waited longer before seeking healthcare during the COVID-19 pandemic compared with the same period before the pandemic. In a study of 44,000 participants conducted in Belgium after the COVID-19 outbreak, the number of people reporting fear of contagion, anxiety or a depressive disorder had increased substantially compared to a survey conducted before pandemic [26].

Similar results have been found in relation to other viruses, with studies analysing health information system data from Liberia, Guinea and Sierra Leone during the Ebola virus outbreak [27–29] demonstrating a significant decrease in the delivery of maternal, child and reproductive health services, interruptions in HIV and tuberculosis testing, and large-scale reductions in vaccine and malaria case management programs. This illustrates that the negative impact on healthcare utilization is not unique to COVID-19, but is more broadly relevant to epidemics of infectiour diseases. Therefore, policymakers and health care providers must design and implement appropriate and effective policies not only for the current pandemic, but also for any future crises to ensure critical healthcare services remain accessible for all diseases.

An interesting finding from the current study was the significant increases seen in the monthly rates of hospitalizations after the COVID-19 outbreak for neoplasm disease, diseases of the digestive system, and pregnancy, childbirth and the puerperium disease. This finding is not consistent with the results of previous studies. For example, Hartnett et al. [11] found that the largest declines in hospitalisations were for abdominal pain and other digestive or abdominal signs and symptoms, compared to our findings of a significant increase of hospitalizations for diseases of the digestive system.

The significant increase in hospitalizations for some disease identified in our study may be partly related to side effects of coronavirus. Previous studies have indicated that gastrointestinal symptoms, anorexia, diarrhea, nausea, and vomiting, abdominal pain and gastrointestinal bleeding are clinical symptoms of COVID-19 thus the increases demonstrated in the current study may be related to the treatment of COVID-19 [30–32]. In addition, these increases may be related to risk factors for hospitalization from COVID-19. For example, a large cohort study in the United States, which included population-level data from 91,412 women, revealed that pregnancy was associated with a significantly increased chance of hospitalization from COVID-19 [33]. Also, the majority of studies have shown that the risk of severe COVID-19 in pregnancy appears to be no greater than for the general population [34–36] and it can affect fear of infection among this groups.

Although the present study provides robust evidence of the association between the COVID-19 pandemic and hospitalization rates in Iran, there were several limitations which should be acknowledged. First, the data in this study were collected in only one region of the country and thus the generalizabity of the study findings are limited. Second, based on the observational period studied, we are not able to examine the longer-term trends in hospitalization rate changes for the diseases included in the study. Thirdly, the study focused only on public sector hospitals as we did not haveaccess to data on hospitalization rates in private and social security hospitals. Thus these results may not be generalizable to all hospitals in the region.

## Conclusion

The current study provides a unique perspective on the impact of COVID-19 on medical admissions in Iran in the months after the COVID-19 outbreak began. It provides insight to health system leaders and public health authorities about groups of patients at continued risk for undertreatment of acute medical illness as a result of the pandemic, which may have harmful health consequences for many patients. Whilst some recovery in monthly hospitalizations was evident, hospitalizations had not returned to pre-COVID-19 rates 17 months after the pandemic began. These results suggest that government policies are needed to fully reverse the disruption to essential healthcare services for non-COVID-19 diseases which has been caused by the redistribution of healthcare resources to the treatment of COVID-19 patients. In addition, appropriate policies should be designed and implemented to reduce fears and anxieties around COVID-19 which have discouraged patients from seeking help from and accessing essential healthcare services.

## Data Availability

The data used for the analysis in this study are available from the corresponding author upon reasonable request.
